# Ten Years of TeleHealth and Digital Healthcare: Where Are We?

**DOI:** 10.3390/healthcare11060875

**Published:** 2023-03-17

**Authors:** Daniele Giansanti

**Affiliations:** Centro Tisp, Istituto Superiore di Sanità, 00161 Roma, Italy; daniele.giansanti@iss.it; Tel.: +39-06-49902701

## 1. Digital Healthcare and TeleHealth

Due to the development of the technological innovation of devices, availability of increasingly performing networks, improvement of the digitization processes, and the push to greater diffusion determined by the COVID-19 pandemic, Digital Healthcare (DH), also referred to as Digital Health [1,2,3,4], and Telehealth (TH), also referred to as Telemedicine [5,6,7,8], have undergone an impressive development in recent years. Technological innovation concerns with sensorization, monitoring, control, and communication electronics. The push to miniaturization of the realization processes and the increasingly performing processing has increased accessibility of portable and/or wearable devices to everyone, and these devices can be used integrated with mobile technology. However, technological innovation also concerns with the mechanics of electronic and computer science integration within mechatronic devices intended for the health domain. Computer science has never delayed development, having a leading role in both the functioning of the devices, and the new and innovative solutions distributed throughout health services. This field is additionally able to rely on the development of new algorithms, based on artificial intelligence (AI). As a result of the technological innovations of information and electronics of communications, networks have become increasingly widespread, accessible, fast, and competitive, regarding both fixed and mobile networks. The digitization processes have become increasingly simplified and inexpensive, and large volumes of data, or commonly, Big Data, have been made available. Access, management, processing, and the exchange of clinical data between professionals (including images, remote monitoring, the exchange of bidirectional information between citizens and health services, and many other processes within the health domain) have become a lot simpler and more routine. The COVID-19 pandemic heavily contributed to the push towards TH and DH. In the need for social distancing and the optimization of care processes, two cardinal elements within the health domain have been identified, in both TH and DH. COVID-19 has significantly facilitated advancement of both the research, and policies and regulations of the TH and DH fields. Following the acute phases of the COVID-19 pandemic, both DH and TH have entered common language in the relationship between citizens and the health system, certainly in the most developed countries.

## 2. TeleHealth and Digital Healthcare Today

With technological evolution, DH and TH have occasionally adapted their field of intervention. DH and TH are two interconnected fields of the health domain, whereby the first includes the latter. DH refers to all the activities encompassing the digitization of medicine and healthcare, whilst TH is an element included in DH, allowing the patients to participate in remote computer-based consulting with doctors, or providing the professionals an opportunity to interact with patients to exchange data information and/or perform collaborative diagnoses.

Beyond TH, today, DH [1,2,3,4,9] includes portable and wearable devices, mobile health, personalized medicine for individuals, health information technology (IT), such as in-hospital information systems and radiology information systems. Digitization is revolutionizing DH owing to the applications in both eHealth and mHealth, and the decision-based support software based on AI.

Currently, TH utilizes a healthcare provider (HCP), avoiding the in-person doctor/specialist interaction [5,6,7,8,10,11]. eHealth utilizes the internet based on personal computer solutions, and mHealth does so based on smartphones, or tablets. This allows real time monitoring of the patient by the HCP, using portable or wearable devices. DH allows for various interactions with a HCP, including cybersecure emails, messages or file exchange, participating in a phone or video call consultation, collaborating with professionals in teleconsulting and decisions regarding diagnosis.

TH, for example, permits: (a) mental health therapy and treatment, (b) dermatology self-monitoring, (c) accessing to clinical results, (c) follow-up after hospital dismissison, (d) management of medical prescriptions, (e) remote rehabilitation and assistance, (f) self-monitoring and self-therapy, in the case of diabetology or cardiology, for example, (g) emergency interventions, and (h) telemonitoring and teleconsulting.

## 3. The Evolution and Trends of the Scientific Production

A simple analysis conducted via Pubmed allows us to make some considerations on the development of TH and DH, as much research appears among scientific publications of the last decade.

A search of studies focusing on TH, with the “telehealth [Title/Abstract]” keyword, was carried out simultaneously with the production of this editorial [12]. The following results were found:

- A total of 12,892 papers, of which 1837 are reviews [13].
- A beginning of scientific dissemination in 1978.
- In the last decade, the number of papers produced was 11,448 [14], equivalent to 88.8% of the entire historical production. Of these papers, 1662 are reviews [15].
- In the most recent period, commencing from the outbreak of the COVID-19 pandemic to date, 8482 papers, equating to 65.8% of the entire scientific production, have been published [16].

A search of studies focusing on DH, with the “digital healthcare [Title/Abstract]” Keyword, was carried out simultaneously with the production of this editorial [17]. The following results were found:

- A total of 340 papers, of which 91 are reviews [18].
- A beginning of scientific dissemination in 2003.
- In the last decade, the number of papers produced was 336 [19], equivalent to 98.8% of the entire historical production. Of these papers, 90 are reviews [20].
- In the most recent period, commencing from the outbreak of the COVID-19 pandemic to date, 265 papers, equating to 77.9% of the entire scientific production, have been published [21].

This brief overview, before all, highlights that DH, formally including TH, has a less voluminous and decidedly more recent history of scientific dissemination, which both follows the consolidation of the upsurge in digital development with mHealth in the health domain, and the evolution and/or adaptation of the terminology.

Furthermore, this overview also shows the great growth that has taken place in the last ten years in scientific production both in DH and in TH. This growth has even become impressive in the three years immediately following the outbreak of the Covid-19 pandemic.

## 4. The Impact of COVID-19 in This Field

In the most recent period, commencing from the outbreak of the COVID-19 pandemic, scientific production has undergone a further acceleration in both fields, regarding technology, employment, and policies and regulation [22,23].

The COVID-19 pandemic has presented a real training ground in this field and an unexpected growth stimulus never experienced before, with the consolidation of ancient areas and the exploration of new areas. Furthermore, even in TH and DH, one can currently count on the use of emerging technologies, such as robotics [24,25,26,27], augmented reality [28], virtual reality [29], and artificial intelligence [30,31]. Moreover, assistive technologies have played a strategic role during the pandemic as they were consolidated in the context of different applications and disabilities relating communication and motion [32,33,34,35,36,37,38,39,40,41].

In addition to the consolidated applications in this sector, new ones have been activated. An application that has exponentially developed in this period has been digital contact tracing for control, monitoring, and epidemic alerting through mobile apps and mHealth solutions [42,43,44,45,46,47,48]. TH and DH have also been integrated in the use of artificial AI tools in the diagnosis and treatment of COVID-19. Digital radiology, digital pathology, and digital dermatology, as brief examples, have explored new potential in health care [49,50,51]. Big Data have played a strategic role in overcoming the pandemic [52]. Assistance for the disabled has tested new solutions [24,25,26,27], such as the social robot, which has played an innovative role as a support and/or mediator between a therapist and a patient [24,25,27]. In this case, the therapist have experienced a radical change in their role. For example, following the pandemic, the professional figure of augmented physiotherapist or digital physiotherapist are being considered. Many mobile apps bordering medical devices and non-medical devices, for physical wellness, including psychological wellness (e.g., gymnastics, walking, nutrition, emotional support, nutrition) have undergone important development [53]. COVID-19 has also stimulated the improvement the use of DH and TH through policy interventions and improvement of regulations, as exemplified by Italy, where the rules of engagement were not initially adequately defined [54]. Approximately a year after the outbreak of the pandemic, the TH and DH sector could rely on 215 search optimization keywords (SEO) reported in [55]. 

COVID-19 has also lead to bottlenecks. The digital divide represents a major obstacle for the use of TH and DH [56,57,58,59,60,61]. Literacy with IT tools (essential for interacting with DH and TH) is often inadequate and opportunities are lost, even in wealthy countries. In the poorest countries, the component of the digital divide also remains due to access to technology, for economic reasons or due to lack of infrastructure.

The regulatory aspects must also be perfected and internationalized. An example of this is the drive towards technological innovation being followed by regulatory consolidation, related to the trustworthiness of the AI regarding digital radiology integrated with AI [62]. The increase in the use of digital technologies with TH and DH also has important implications for cybersecurity, which must be particularly considered, even in emerging sectors, such as the use of mobile technologies and robotics in healthcare (for example, social robotics), where new risks are present for professionals and citizens [27,53,63].

Attention to strategic aspects of remote interaction must be maximized, taking into account all phases of the connection, even the preliminary aspects, such as informed consent, with its important legal and administrative implications [64]. It will also be necessary to make an optimal use of the opportunities that DH offers with the integration of Big Data, by deriving the maximum benefit from this and inheriting the fruitful experience in the field of digital radiology [65].

## 5. Conclusions and Final Reflection

In the last decade, DH and TH have undergone a rapid acceleration in terms of application of use, as documented by the growth of the number of scientific publications. In the period following the outbreak of the pandemic, this growth has been impressive. New applications, additionally integrated with AI, have had an important development in diagnostics, therapy, assistance, epidemiology, and other sectors. Bottlenecks have also emerged in this recent period, such as: (a) the digital divide into the two components due to literacy and access to infrastructure, (b) the adjustment of the regulations, which must remain updated with technological innovation and the trustworthiness of the AI, and (d) cybersecurity applied to new devices operating in the health domain.

It is necessary to start from the recently consolidated experiences and from the problems persisting in TH and DH, and make a map point in scientific research. Researchers and stakeholders have a strategic role in this. With the special issue opened on the occasion of the tenth anniversary of the healthcare journal [66] (https://www.mdpi.com/journal/healthcare/special_issues/4R7KYJ9CAJ accessed on 31 January 2023), we intend to make a contribution in this direction.
